# Supplementary material for: Pooled Saliva Specimens for SARS-CoV-2 Testing
Source: medRxiv. 2020 Oct 5:2020.10.02.20204859. Preprint. [Version 1] doi: 10.1101/2020.10.02.20204859 (PMC7553188; doi:10.1101/2020.10.02.20204859)
Supplement: Supplement 1 [file media-1.pdf]

Pooled Saliva Specimens for SARS-CoV-2 Testing

Barat et al. Supplementary Data

Table S1: Summary of studies evaluating saliva as a specimen for SARS-CoV-2 testing

Reference, First Author	Characteristics	Collection Method	Total Subjects	Total Positive	Sensitivity/ PPA (%) SAL/ NP	Specificity / NPA (%) SAL/NP	Testing Platform and Notes
Jamal (1)	All known positive	Saliva<br><br>NP swab	91	72	72 SAL<br><br>89 NP	-	Allplex™ 2019-nCoV Assay(100T) (Seegene Inc, Seoul, Korea)<br><br>Saliva + PBS
Nagura-Ikeda (2)	15 asymptomatic,<br><br>88 symptomatic<br><br>All known positive	Saliva<br><br>OP/NP for diagnosis	103	103	SAL<br><br>82 LDT<br><br>81 COBAS<br><br>71 RT-LAMP	-	RT-qPCR LDT, cobas SARS-CoV-2 high-throughput system, RT-LAMP[1]<br><br>Saliva + PBS
Wyllie (3)	In-patient<br><br><br>Asymptomatic health-care workers	Saliva upon waking prior to food/water intake<br><br>NP swab<br><br>Self- collected NP for asymptomatic[2]	70<br><br><br>495	70<br><br><br>9	81 SAL<br>71 NP<br><br>Only 9 positive samples for comparison	-	CDC Assay<br><br>Viral load higher in saliva in patients

Migueres (4)	17 Asymptomatic<br><br>27 Symptomatic<br><br>9 Hospitalized	Saliva<br><br>NP swab	123	44	88 SAL asymptomatic<br><br>95 SAL symptomatic<br><br>93 NP	-	Panther Fusion™ module (Hologic)
Skolimowska (5)	Symptomatic healthcare workers<br><br>5 pediatric patients (<18 years)	Saliva<br><br>Combined OP/NP swab comparison	132	18	83 SAL	99 SAL	Roche, AusDiagnostics, ThermoFisher, Abbott<br><br>cobas ® PCR medium
Pasomsub (6)	Symptomatic	Saliva<br><br>NP and throat swab	200	19	84 SAL	99 SAL	SARS-CoV-2 Nucleic Acid Diagnostic Kit (Sansure, Changsha, China)<br><br>CFX96 Real-Time Detection System (Bio-Rad)
Williams (7)	Screening clinic	Saliva<br><br>NP swab	522	39	85 SAL	98 SAL	Coronavirus Typing (8-well) assay, AusDiagnostics)<br><br>Higher viral load in NP
Zhu (8)		Saliva<br><br>Respiratory tract sample (NP /OP swab)	944	442	86 SAL	97 SAL	

Landry (9)	Symptomatic out-patient	Saliva<br><br>NP swab	124	35	86 SAL<br><br>94 NP	98 SAL	CDC Assay<br><br>1/3 saliva samples were thick
Rao (10)	Asymptomatic adult male, 8-10 day after tested positive<br>SAL from deep in throat upon waking prior to food/water intake	Saliva<br><br>NP swab	217	160	93 SAL<br><br>53 NP	-	One-step RT-PCR , Real-Q 2019 nCoV detection kit (Biosewoom, Inc, South Korea)<br><br>Higher viral load in saliva
Hanson (11)	Drive-thru test center	Saliva<br><br>NP swab	354	81	94 SAL	98 SAL	Hologic Aptima SARS-CoV-2 transcription mediated amplification (TMA) assay<br><br>Saliva + Universal transport media
McCormick-Baw (12)	Emergency department	Saliva<br><br>NP swab	156	50	96 SAL	99 SAL	Cepheid Xpert Xpress SARS27<br>CoV-2 PCR test
Procop (13)	Screening Center	Elicited cough and saliva<br><br>NP swab	216	38	100 SAL PPA	99 SAL NPA	Aptima™ SARS-CoV-2 transcription mediated amplification assay (Panther™ System, Hologic)

Azzi (14)	Confirmed COVID-19 patients with severe or very severe disease	Saliva (drooling technique) Or Endotracheal tube<br><br>NP swab	25	25	100 SAL	-	Abi Prism 7000 sequence detection system (Applied Biosystems)<br><br>QIAmp Viral RNA mini kit (Qiagen)<br><br>Luna Universal qPCR Master Mix (New England BioLab)
Iwasaki (15)	Symptomatic	Saliva<br><br>NP swab	76	9	97 Overall percent agreement	-	One-Step Real-Time RTPCR Master Mixes and tepOnePlus Real Time PCR System (Thermo Fisher Scientific)
Uwamino (16)	32 hospitalized patients<br><br>115 symptomatic staff	Saliva<br><br>NP swab	147	32	87 Overall concordance (SAL & NP)<br><br>96 overall concordance within first 10 days	-	LightCycler96 (Roche, Basel, Switzerland)<br><br>2019 Novel Coronavirus Detection Kit (Shimaszu, Kyoto, Japan)
Byrne (17)	Emergency Department<br><br>In-patient symptomatic	Saliva<br><br>Nasal and throat swab	110	14	86 SAL	-	QIAmp Viral RNA Mini Kit (QIAGEN)<br>Genesig Real-time Coronavirus COVID-19 PCR<br>Similar viral load

[1]RT-LAMP : Reverse transcription-loop-mediated isothermal amplification

[2]All NP/OP healthcare collected except when noted

Table S2: Study Participant Characteristics

	ED	Drive- Through	Total
Number of Participants	69	380	449
Gender, Male N (%)	23 (33%)	159 (42%)	182 (41%)
Age, Range (median)	21-88 (46)	21-75 (42)	21-88 (42)

Figure S1.

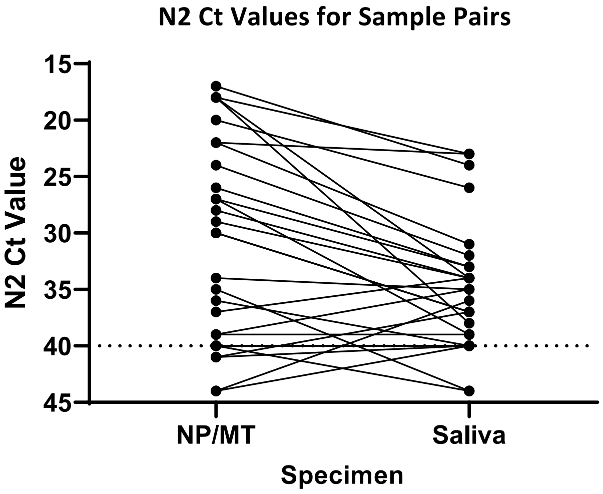

Figure S1. N2 Cycle threshold (Ct) values for paired NP/MT and SAL samples (29 pairs). Pairs are connected by a line. The N2 Ct was set to 40 for samples in which N2 was not detected, indicating a negative result for SARS-CoV-2 RNA. Horizontal dashed line is at Ct=40, the assay cut off. P-value <0.05 calculated by Wilcoxon matched- pair signed rank test.

Figure S2.

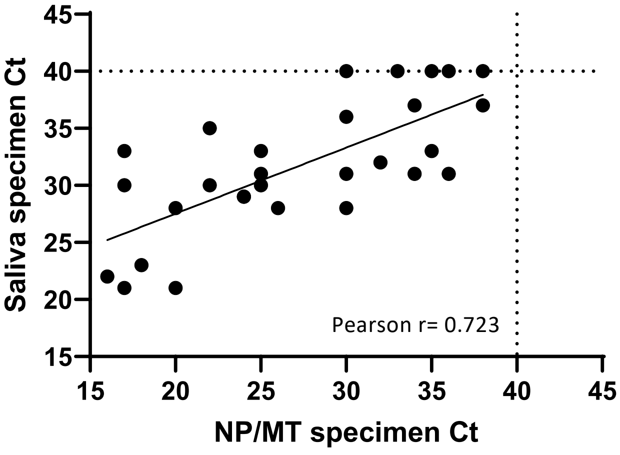

Figure S2. Comparison of Cycle Threshold (Ct) values for N1 for NP versus SAL specimens (29 pairs). Analysis demonstrates moderately good correlation between NP Ct values and Ct values for matched SAL samples. R and p values determined by Pearson correlation and graphically represented by linear regression. Dotted line indicates the limit of detection. P-value <0.001.

Figure S3 (A-B)

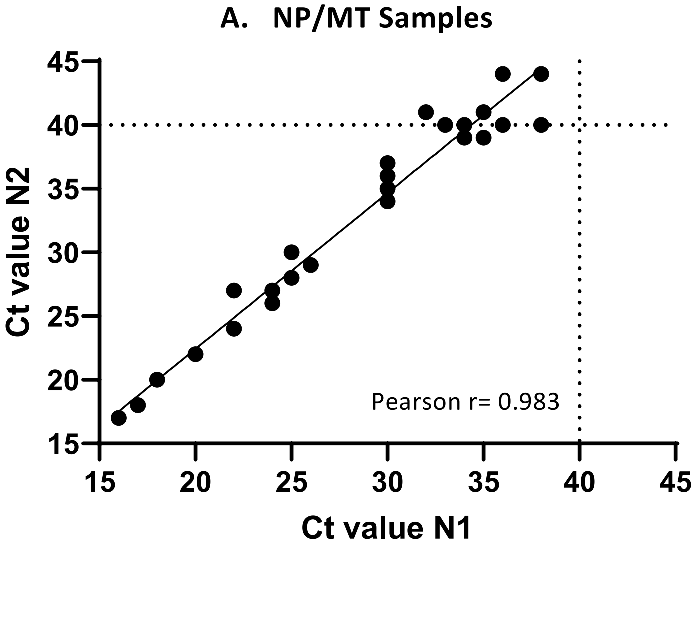

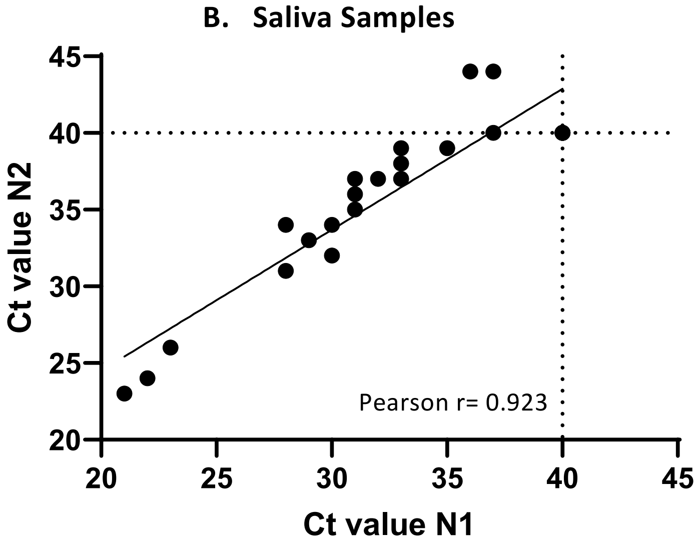

Figure S3 (A-B). Concordance between SARS-CoV-2 detection in 29 samples of each type using CDC N1 primer and probe sets. R and p values determined by Pearson correlation and graphically represented by linear regression. Dotted line indicates the limit of detection. P-value <0.001

Figure S4 (A-C)

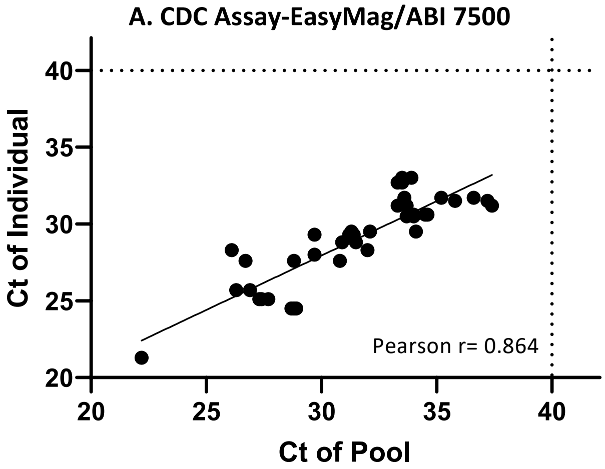

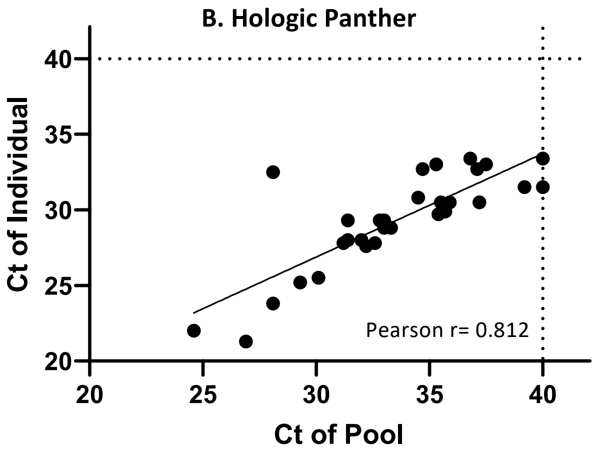

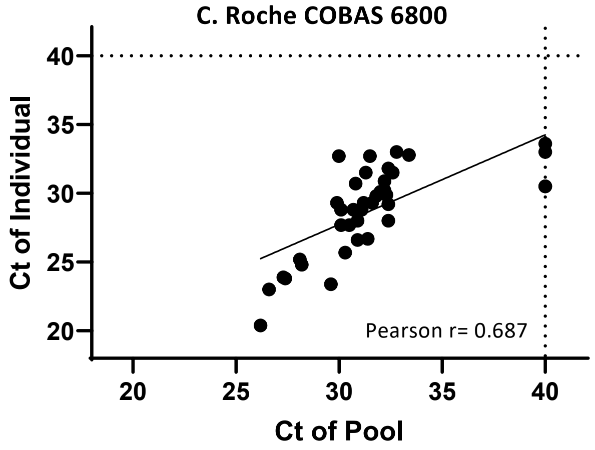

Figure S4 (A-C). Relationship between Ct values of individual and pooled SAL samples. R and p values determined by Pearson correlation and graphically represented by linear regression. Dotted line indicates the limit of detection. P-value<0.001

References

1. Jamal AJ, Mozafarihashjin M, Coomes E, Powis J, Li AX, Paterson A, Anceva-Sami S, Barati S, Crowl G, Faheem A, Farooqi L, Khan S, Prost K, Poutanen S, Taylor M, Yip L, Zhong XZ, McGeer AJ, Mubareka S, Toronto Invasive Bacterial Diseases Network C-I. 2020. Sensitivity of nasopharyngeal swabs and saliva for the detection of severe acute respiratory syndrome coronavirus 2 (SARS-CoV-2). Clin Infect Dis doi:10.1093/cid/ciaa848.

2. Nagura-Ikeda M, Imai K, Tabata S, Miyoshi K, Murahara N, Mizuno T, Horiuchi M, Kato K, Imoto Y, Iwata M, Mimura S, Ito T, Tamura K, Kato Y. 2020. Clinical Evaluation of Self-Collected Saliva by Quantitative Reverse Transcription-PCR (RT-qPCR), Direct RT-qPCR, Reverse Transcription-Loop-Mediated Isothermal Amplification, and a Rapid Antigen Test To Diagnose COVID-19. J Clin Microbiol 58(9):e01438-20. doi: 10.1128/JCM.01438-20.

3. Wyllie AL, Fournier J, Casanovas-Massana A, Campbell M, Tokuyama M, Vijayakumar P, Warren JL, Geng B, Muenker MC, Moore AJ, Vogels CBF, Petrone ME, Ott IM, Lu P, Venkataraman A, Lu-Culligan A, Klein J, Earnest R, Simonov M, Datta R, Handoko R, Naushad N, Sewanan LR, Valdez J, White EB, Lapidus S, Kalinich CC, Jiang X, Kim DJ, Kudo E, Linehan M, Mao T, Moriyama M, Oh JE, Park A, Silva J, Song E, Takahashi T, Taura M, Weizman OE, Wong P, Yang Y, Bermejo S, Odio CD, Omer SB, Dela Cruz CS, Farhadian S, Martinello RA, Iwasaki A, Grubaugh ND, et al. 2020. Saliva or Nasopharyngeal Swab Specimens for Detection of SARS-CoV-2. N Engl J Med doi:10.1056/NEJMc2016359.

4. Migueres M, Mengelle C, Dimeglio C, Didier A, Alvarez M, Delobel P, Mansuy JM, Izopet J. 2020. Saliva sampling for diagnosing SARS-CoV-2 infections in symptomatic patients and asymptomatic carriers. J Clin Virol 130:104580 doi: 10.1016/j.jcv.2020.104580.

5. Skolimowska K, Rayment M, Jones R, Madona P, Moore LSP, Randell P. 2020. Non-invasive saliva specimens for the diagnosis of COVID-19: caution in mild outpatient cohorts with low prevalence. Clin Microbiol Infect doi:10.1016/j.cmi.2020.07.015.

6. Pasomsub E, Watcharananan SP, Boonyawat K, Janchompoo P, Wongtabtim G, Suksuwan W, Sungkanuparph S, Phuphuakrat A. 2020. Saliva sample as a non-invasive specimen for the diagnosis of coronavirus disease 2019: a cross-sectional study. Clin Microbiol Infect doi:10.1016/j.cmi.2020.05.001.

7. Williams E, Bond K, Zhang B, Putland M, Williamson DA. 2020. Saliva as a Noninvasive Specimen for Detection of SARS-CoV-2. J Clin Microbiol 58(8):e00776-20 doi: 10.1128/JCM.00776-20.

8. Zhu J, Guo J, Xu Y, Chen X. 2020. Viral dynamics of SARS-CoV-2 in saliva from infected patients. J Infect 81:e48-e50 doi: 10.1016/j.jinf.2020.06.059.

9. Landry ML, Criscuolo J, Peaper DR. 2020. Challenges in use of saliva for detection of SARS CoV-2 RNA in symptomatic outpatients. J Clin Virol 130:104567 doi: 10.1016/j.jcv.2020.104567.

10. Rao M, Rashid FA, Sabri F, Jamil NN, Zain R, Hashim R, Amran F, Kok HT, Samad MAA, Ahmad N. 2020. Comparing nasopharyngeal swab and early morning saliva for the identification of SARS-CoV-2. Clin Infect Dis doi:10.1093/cid/ciaa1156.

11. Hanson KE, Barker AP, Hillyard DR, Gilmore N, Barrett JW, Orlandi RR, Shakir SM. 2020. Self-Collected Anterior Nasal and Saliva Specimens versus Healthcare Worker-Collected

Nasopharyngeal Swabs for the Molecular Detection of SARS-CoV-2. J Clin Microbiol doi:10.1128/JCM.01824-20.

12. McCormick-Baw C, Morgan K, Gaffney D, Cazares Y, Jaworski K, Byrd A, Molberg K, Cavuoti D. 2020. Saliva as an Alternate Specimen Source for Detection of SARS-CoV-2 in Symptomatic Patients Using Cepheid Xpert Xpress SARS-CoV-2. J Clin Microbiol 58(8):e01109-20. doi: 10.1128/JCM.01109-20.

13. Procop GW, Shrestha NK, Vogel S, Van Sickle K, Harrington S, Rhoads DD, Rubin BP, Terpeluk P. 2020. A Direct Comparison of Enhanced Saliva to Nasopharyngeal Swab for the Detection of SARS-CoV-2 in Symptomatic Patients. J Clin Microbiol doi:10.1128/JCM.01946-20.

14. Azzi L, Carcano G, Gianfagna F, Grossi P, Gasperina DD, Genoni A, Fasano M, Sessa F, Tettamanti L, Carinci F, Maurino V, Rossi A, Tagliabue A, Baj A. 2020. Saliva is a reliable tool to detect SARS-CoV-2. J Infect 81:e45-e50 doi: 10.1016/j.jinf.2020.04.005.

15. Iwasaki S, Fujisawa S, Nakakubo S, Kamada K, Yamashita Y, Fukumoto T, Sato K, Oguri S, Taki K, Senjo H, Sugita J, Hayasaka K, Konno S, Nishida M, Teshima T. 2020. Comparison of SARS-CoV-2 detection in nasopharyngeal swab and saliva. J Infect 81:e145-e147 doi: 10.1016/j.jinf.2020.05.071.

16. Uwamino Y, Nagata M, Aoki W, Fujimori Y, Nakagawa T, Yokota H, Sakai-Tagawa Y, Iwatsuki-Horimoto K, Shiraki T, Uchida S, Uno S, Kabata H, Ikemura S, Kamata H, Ishii M, Fukunaga K, Kawaoka Y, Hasegawa N, Murata M. 2020. Accuracy and stability of saliva as a sample for reverse transcription PCR detection of SARS-CoV-2. J Clin Pathol doi:10.1136/jclinpath-2020-206972.

17. Byrne RL, Kay GA, Kontogianni K, Aljayyoussi G, Brown L, Collins AM, Cuevas LE, Ferreira DM, Fraser AJ, Garrod G, Hill H, Hughes GL, Menzies S, Mitsi E, Owen SI, Patterson EI, Williams CT, Hyder-Wright A, Adams ER, Cubas-Atienzar AI. 2020. Saliva Alternative to Upper Respiratory Swabs for SARS-CoV-2 Diagnosis. Emerg Infect Dis 26(11) doi: 10.3201/eid2611.203283.
